# LERMS: A Low-Latency and Reliable Downlink Packet-Level Encoding Transmission Method in Untrusted 5GA Edge Network

**DOI:** 10.3390/e25070966

**Published:** 2023-06-21

**Authors:** Zhongfu Guo, Xinsheng Ji, Wei You, Mingyan Xu, Yu Zhao, Zhimo Cheng, Deqiang Zhou, Lingwei Wang

**Affiliations:** 1 Department of Next-Generation Mobile Communication and Cyber Space Security, Information Engineering University, Zhengzhou 450001, China; 2 National Digital Switching System Engineering and Technological Research and Development Center, Zhengzhou 450000, China; 3 Purple Mountain Laboratories: Networking, Communications and Security, Nanjing 211111, China

**Keywords:** 5G-A core network, robust concurrent multipath transfer, interface diversity edge network, raptor codes, security

## Abstract

The increasing demand for end-to-end low-latency and high-reliability transmissions between edge computing nodes and user elements in 5G Advance edge networks has brought new challenges to the transmission of data. In response, this paper proposes LERMS, a packet-level encoding transmission scheme designed for untrusted 5GA edge networks that may encounter malicious transmission situations such as data tampering, discarding, and eavesdropping. LERMS achieves resiliency against such attacks by using 5GA Protocol data unit (PDU) coded Concurrent Multipath Transfer (CMT) based on Lagrangian interpolation and Raptor’s two-layer coding, which provides redundancy to eliminate the impact of an attacker’s malicious behavior. To mitigate the increased queuing delay resulting from encoding in data blocks, LERMS is queue-aware with variable block length. Its strategy is modeled as a Markov chain and optimized using a matrix method. Numerical results demonstrate that LERMS achieves the optimal trade-off between delay and reliability while providing resiliency against untrusted edge networks.

## 1. Introduction

In the era of 5G [1], edge computing provides customized computing services for data-intensive [2] and time-sensitive [3] applications such as healthcare [4] and traffic management [5]; edge networks (ENs) [6] are designed to address the challenges of centralized cloud computing and unreliable communications [7,8]. 5G-Advanced networks [9] are expected to offer more powerful capabilities, including wide coverage [10], low latency [11], and highly reliable transmission [12].

The EN transmits important and sensitive data related to safety [13], but it is closer to users and vulnerable to wireless eavesdropping [14], network-layer attacks [15], and tampering [16]. The edge network often finds itself in a predicament of weak protection capabilities and urgent security needs.

Previous studies have focused on addressing these pressing demands [17], and researchers have conducted extensive studies on point-to-point (UE-gNB) security at both the physical layer [18] and MAC-layer [19]. Providing secure end-to-end (UE-DN) transmission in an untrusted network incurs additional performance and resource overheads [20]. Commonly used confidentiality protection schemes include 5G voice communication using IPSec encryption [21], control plane TLS authentication [22], and secret-sharing proposed by Shamir et al. [23]. In the secret-sharing scheme, each share is delivered by a different courier to the recipient, providing an information-theoretic provable confidentiality transmission scheme.

**Figure 1:**
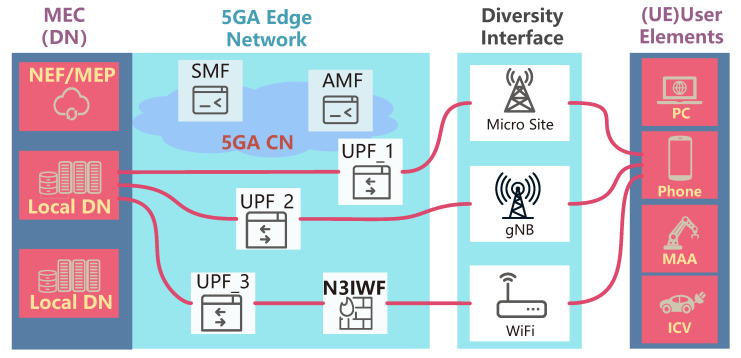
End-to-end redundant transmission in edge networks: A schematic illustration of MPEN utilizing redundant PDU sessions in an edge network with diversity interface for concurrent multipath transmission.

The enhanced core network (ECN) proposed by 3GPP in ([1], clause 5.33), as shown in Figure 1, could establish multiple PDU sessions (MP), which can provide end-to-end reliable communication by redundancy transmission. The multiplex transport protocol stack is deployed on both the data network (DN) and user elements (UEs). Additionally, the high-layer (above the IP layer [1]) splits and aggregates the service flows. However, implementing concurrent multipath transmission (CMT) in an untrusted MPEN faces many challenges, including increased attack surface due to MPEN [17], additional delays due to asynchronous delay and bandwidth [24,25], making it not feasible to interact with the PDU bearer from the perspective of the 5G session mechanism [26].

In this paper, we aim to design a low-latency, high-reliability transmission scheme suitable for untrusted edge networks. Randomly arriving source data packets are buffered and queued at the sending end before transmission. This article continues previous work [27] and uses Raptor coding for efficient CMT. We have introduced a Lagrangian interpolation coding [28] scheme to realize CMT based on secret sharing. Different coding parameters can be adjusted for different resilience levels, which is suitable for an untrusted network environment, as the concatenated encoder provides reliability and robustness against malicious behavior. Encoding is performed at the granularity of data packets. Longer encoding block length can ensure reliability, but the corresponding queuing delay will increase [29]. The length of encoding blocks must be carefully considered to balance delay and reliability. We use a Markov decision process to formulate the optimization problem and construct it as a matrix-based linear programming to obtain the optimal variable-length coding strategy. The contributions of this paper are as follows:

- Proposes LERMS (LERMS is short for (Lagrangian–polynomial and Raptor Encoder concurrent Multiple-pdu-Sessions transmission)), which is the first concatenated encoder scheme that is suitable for untrusted edge network environments. LERMS provides secure, reliable downlink transmission capabilities in the face of an untrusted network, such as transmission failures, data theft, and malicious tampering.
- A queue-aware variable block length encoding scheme is designed and optimized using a matrix-based approach to minimize queuing delay while ensuring reliability.
- Proposes a multi-service flow aggregation transmission scheme that reduces the probability of data packet random idle filling, ensuring security when the flow is small, and improves the transmission efficiency of the edge network.

This paper is organized as follows: Section 2 presents the design of the secure transmission method and flow aggregation transmission scheme as preliminary work for 5GA LERMS. Section 3 describes the system model. In Section 4, the trade-off between delay and reliability for the LERMS strategy is demonstrated. Section 5 presents numerical results. This paper concludes in Section 6, with the future research directions proposed. Table 1 summarizes the important variables used throughout the paper.

## 2. 5GA LERMS Preliminary

We consider the business scenario in the edge network, as illustrated in Figure 1. The UE subscribes to the edge computing service, and the edge network that transmits is responsible for transmitting the downlink service flow to the UE. The downlink service flow requires low delay and high reliability in transmission. With abundant transmission resources, the 5GA network allows for the establishment of multiple PDU sessions to carry the downlink traffic. To ensure the secure transmission of the downlink traffic in an untrusted network environment, this section begins by analyzing the key performance indicators in the edge network environment. We then propose a multiplex transmission scheme based on the concept of secret sharing, which provides feasibility for secure transmission. Finally, we discuss a joint coding scheme for downlink traffic in the core network.

### 2.1. 5GA Edge Network Requirements Analysis and Challenges

As an emerging network architecture that provides high-performance communication between terminals (UE) and edge computing nodes (DN), the 5G Advance (5GA) edge network [6] aims to meet key requirements such as low latency, high reliability, and security in the 5GA mobile network. In this section, we will analyze these requirements in detail and discuss the challenges of implementing them in an untrusted network environment.

**Latency** (*L*). In classical transmission systems, the overall transmission latency comprises propagation latency ($t_{p}$), signifying the physical delay of signals within the transmission medium, and transmission latency ($t_{c}$), determined by $t_{c}=W/\phi$, where *W* represents the bandwidth and ϕ is the transmitted data volume. However, factors such as congestion and bit errors prevent the total latency from being a simple sum of $t_{p}$ and $t_{c}$, causing transmission latency fluctuations within a specific range [30,31]. In coded transmission systems, end-to-end transmission delay must be reconsidered. Data are coded and transmitted in block units. Current application layer data enter the cache and queue up, waiting for the previous generation of coded data blocks to finish transmission [32]. Consequently, the end-to-end transmission delay is defined as $\overset{↔}{\;T}$ = *T* + *t_en_* + *t_de_* + *t_q_*, encompassing codec delay and queuing delay. This paper’s low latency discussion primarily centers on *t_q_* and *t_c_*.

**Reliability** (*R*). In this paper, we consider each PDU session as a packet erasure channel, denoted by $\varepsilon_{\upsilon }^{j}=\left[\varepsilon_{1},\cdots ,\varepsilon_{j}\right]$ to represent the erasure probability of each PDU session [27]. High reliability refers to the transmission robustness against higher erasure probabilities. In other words, when a generation of source data packets departs from the sender within a specified time, and after applying redundancy/retransmission/coding and other reliable transmission techniques, we call it reliable transmission [17]. However, such losses may still be manipulated by malicious nodes, such as attackers, or intercepted by eavesdroppers. We define the total erasure probability as $E_{N_{L}}=\sum_{j=1}^{N_{L}}\gamma_{\upsilon }^{j}\varepsilon_{j}$, where $\gamma_{\upsilon }^{j}={\left[\gamma_{1},\cdots ,\gamma_{j}\right]}^{T}$ represents the traffic load weight of each path, with $\sum_{j=1}^{N_{L}}\gamma_{\upsilon }^{j}=1$, and $0\leq EN_{L}<1$. We refer to the current MPEN as $E_{N_{L}}$-Level reliability.

**Security**. Security performance refers to the robustness of maintaining correct data delivery in the face of attackers [33]. Unlike traditional trust management [34] to ensure system security, our focus is on enhancing communication security through the lens of information theory. It takes into account active attackers who may tamper with or pollute the data received by the destination, and passive attackers who attempt to eavesdrop on and steal the data. We consider rational attackers who target only a subset of PDU sessions in the edge network rather than all of them. By treating all malicious actions as packet errors, we can employ redundant coding techniques to protect and repair the corrupted or stolen data, thereby enhancing the security performance in the edge network environment. This approach ensures the integrity and confidentiality of data transmission, even in the presence of adversaries targeting edge networks. Therefore, we can regard the redundancy of multi-path coded transmission as the security level, and we can simply refer to the edge network where $N_{L}$ PDU sessions are transmitted as $N_{L}$-level secure transmission.

To sum up, service flows carried by edge networks require low-latency, high-reliability transmission with elastic capabilities in an untrusted network environment. However, achieving these requirements in practice is not easy. Due to the untrustworthy characteristics of edge networks, such as congestion, bit errors, and malicious attacks, more advanced transmission schemes and technologies need to be researched and designed to meet these challenges and ensure safe and efficient data transmission in edge networks.

### 2.2. A Secure Transmission Method

In this section, we analyze the transfer security of secret sharing. Secret sharing is a method of splitting data and generating multiple pieces of data through Lagrangian interpolation to transmit data over untrusted multiple paths. This approach is designed to achieve a level of security that protects data from potential attackers while providing protection against partial data-tampering attacks.

The core idea of secret sharing technology is to decompose the original data into multiple derivative pieces of data using the Lagrangian interpolation method. Let us suppose we need to transmit the original data D and decompose its encoding into n pieces of derivative data, among which any *k* shares of data (k≤n) are sufficient to reconstruct the original data (In the secret-sharing technique [23,35], the original data is split into multiple parts, referred to as “shares” (derived data). These shares in LERMS are then transmitted through multiple paths, with each path carrying one share of the data). First, a polynomial of degree k−1 is generated using Lagrange interpolation:

(1) $$f\left(x\right)=a_{0}+a_{1}x+a_{2}x^{2}+\cdots +a_{\left(k-1\right)}x^{\left(k-1\right)}$$

where $a_{0}=D,a_{1},a_{2},\cdots ,a_{\left(k-1\right)}$ are randomly selected coefficients. Then, we calculate the value of f(x) at *n* different points to generate *n* derivative data:

(2) $$D_{i}=f\left(x_{i}\right),i=1,2,\cdots ,n$$

To reconstruct the original data, they can be calculated by Lagrangian interpolation:

(3) $$D=L\left(0\right)=\sum_{i=1}^{k}l_{i}\left(0\right)D_{i}$$

$l_{i}\left(x\right)$ is a Lagrangian basis function that satisfies $l_{i}\left(x_{j}\right)=\delta_{ij}$ $\mathrm{that}\;\mathrm{is},\;\mathrm{when}\;i=j,\;l_{i}\left(x_{j}\right)=1;\;\mathrm{when}\;i\neq j,\;l_{i}\left(x_{j}\right)=0,\;\mathrm{defined}\;\mathrm{as}:$

(4) $$l_{i}\left(x\right)=\prod_{j\neq i}^{k}\frac{\left(x-x_{j}\right)}{\left(x_{i}-x_{j}\right)},j\neq i,j=1,2,\cdots ,k$$

In the edge network, each PDU session is considered an independent link provider, with a probability of being infiltrated by malicious behavior. The secret sharing transmission scheme based on Lagrangian interpolation in this scenario offers the following properties: **Information security**: In order to restore the original data through Lagrangian interpolation, the attacker must obtain at least *k* shares of derived data simultaneously, which increases the difficulty of their job and enhances information security. **Integrity**: Since the original data *D* is only dependent on the linear combination of the derived data $D_{i}$, an attacker cannot change the value of the original data by tampering with part of the derived data unless they control at least *k* shares of the derived data at the same time. **Reliability**: As any *k* shares of derived data can be used to restore the original data, the reliability of the data can be guaranteed even if part of the derived data is lost or damaged during transmission.

**Figure 2:**
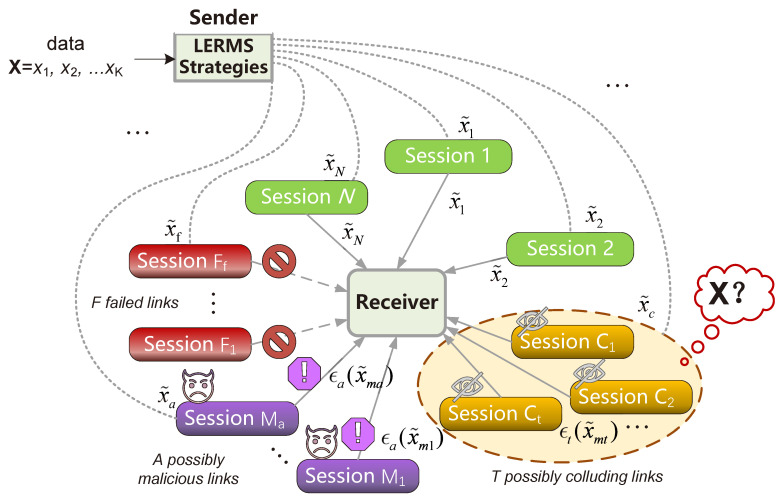
This diagram illustrates the transmission model (MPEN) with untrusted paths, which is an extended application of the problem model in [28]. The focus of this paper is to transmit X from the sender to the receiver through $N_{L}$ PDU sessions with low-latency and high-reliability characteristics, despite facing multiple threats. By carefully designing the LERMS strategies, the receiver can collect whole data from a subset of PDU sessions’ messages, even in the presence of failed links ($F_{1},\cdots ,F_{f}$) and malicious links ($M_{1},\cdots ,M_{a}$), while also ensuring data privacy from colluding links ($C_{1},\cdots ,C_{t}$).

To summarize, the CMT of secret sharing is robust and provides security guarantees for data transmission. We aim to further explain the resilient delivery methods that our proposed scheme LERMS can provide by identifying the types of security challenges it addresses. As shown in Figure 2, an untrusted edge network may contain invalid links that result in complete data loss, malicious links that tamper with or forge data, or colluding attackers that steal or modify data to launch a Byzantine attack. These malicious behaviors can be viewed as code errors that need to be corrected. Drawing from the concept of redundancy checks, we adjust the generation strategy of derived data to tolerate different types of malicious behaviors during transmission. In the next section, we will discuss more specific secure transmission schemes in the double-layer concatenated encoder.

### 2.3. Downlink Multi-Service Stream Joint Coding

In an untrusted edge network environment, the data transmission of edge computing nodes faces numerous challenges, including transmission efficiency, privacy, security, and reliability [36]. Due to the minimum limit of the length of the data flow resulting from the splitting of path transmission data and security coding, padding short packets is often necessary to meet the requirement. However, this can lead to a waste of resources.

To tackle these issues and demonstrate the demand and advantages of aggregating and transmitting multiple downlink service flows, we have developed an approach based on the current core network data transmission process. This approach enables the efficient transmission of aggregated data flows while preserving data privacy. As an example, we introduce an illustrative scenario called Single-Owner Multi-Device Data Transmission with Joint Encoding (SOMD-JE), which serves to further prove the value and practical effect of aggregated transmission in 5GA edge networks.

**Figure 3:**
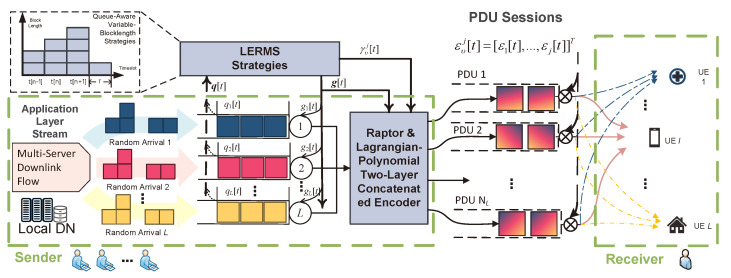
System model: downlink-server flow transmission with joint encoding.

In the SOMD-JE scenario, we jointly encode multiple downlink service flows, which may belong to different devices but share the same owner. By analyzing the data characteristics of these flows, we found that aggregating and transmitting them does not lead to privacy leakage, and instead, the SOMD-JE improves transfer efficiency through server flows aggregation. For instance, in a smart home scenario, users’ mobile phones, smart screens, VR devices, and elderly health monitoring devices all belong to the same owner (As shown in Figure 3, the receiver has multiple devices including terminals, medical monitoring devices, and smart home devices), so aggregated data flows can be transmitted without worrying about privacy leaks. Similar settings exist on hospital wards and factory floors.

This flows aggregation approach overcomes the waste of resources caused by traditional padding methods, makes full use of edge network transmission resources, and ensures safe, reliable, and efficient transmission while maintaining data privacy. The specific scheme for aggregating and transmitting downlink service flows needs to be adaptively adjusted within the current core network data transmission process. For further details, please refer to Appendix A.

## 3. System Model

### 3.1. PDU Session Queuing and Encoding Transmission Model

For data packets arriving from the application layer, we assume that the arrival of downlink data packets of different users is completely independent and identically distributed (i.i.d.). Let $\Lambda_{l}\left[t\right]$ denote the number of Ps arriving in the *t*-th timeslot for user *l* (l=1,2,⋯,L). Given that the maximum value of $\Lambda_{l}\left[t\right]$ is *N*, the probability distribution of $\Lambda_{l}\left[t\right]$ for user *l* is expressed as $\lambda ={\left[\lambda_{l}^{0},\lambda_{l}^{1},\cdots ,\lambda_{l}^{N}\right]}^{T}$, where $\lambda_{l}^{n}=P\left\{\Lambda_{l}\left[t\right]=n\right\}$ denotes the probability of user *l* receiving *n* packets in the *t*-th timeslot. The average arrival rate is defined as $\bar{\Lambda_{l}}=\sum_{n=0}^{N_{\Lambda }}n\lambda_{l}^{n}$.

For each user, a buffer with a size of Z, randomly arriving packets are accumulated, and $g_{l}\left[t\right]$ packets are selected from the user *l*’s cache once the encoding of the previous block of packets is completed. Therefore, $q_{l}\left[t\right]\in Z=\left\{0,1,\cdots ,Z\right\}$, in the [t+1]-th timeslot, $q_{l}\left[t\right]$ evolves as (we define ${\left(x\right)}^{*}=\max\left\{x,0\right\}$)

(5) $$q_{l}\left[t+1\right]=\min\left\{{\left(q_{l}\left[t\right]-g_{l}\left[t\right]\right)}^{*}+\Lambda_{l}\left[t+1\right],Z\right\}.$$

We assume that the size of the data block $g_{l}\left[t\right]$ selected for each user and each generation of encoding does not exceed *B*, i.e., $g_{l}\left[t\right]\in N=\left\{0,1,\cdots ,B\right\}$. The feasible region of each user’s cache queue length $q_{l}\left[t\right]$ is given by $q_{l}\left[t\right]\in Z$. Under the block length selection strategy N, $N\left(q\right)=\{g\in N|{\left(0,q-B+N_{\Lambda }\right)}^{*}\leq g\leq \min\left(q,B\right)\}$, where the feasible range N(q) guarantees that each user’s sending buffer queue will not underflow or overflow, we also have $B\geq N_{\Lambda }$ which ensures that the system will not be congested.

Under the LERMS strategies, at time slot *t* we encode and transmit a generation of $g_{\sigma }\left[t\right]$ data packets (Ps) (Assuming that all packets have the same length and carry ϕ bit information), where $g_{\sigma }\left[t\right]=\Sigma_{l=1}^{L}g_{l}\left[t\right]$. The specific encoder process will be described in detail in the next subsection. LERMS choose $N_{L}$ PDU sessions for transmission, and the transmission vector is denoted as $\gamma_{\upsilon }^{j}={\left[\gamma_{1},\cdots ,\gamma_{j}\right]}^{T}$, where $\gamma_{\upsilon }^{j}$ indicates which channels are used for transmission. The output share of Lagrangian coding is also determined based on the number of channels. In summary, we express the LERMS strategies action as $\left(g_{l},\gamma_{\upsilon }^{j}\right)$, which changes in units of time slots, i.e., $\left(g_{l}\left[t\right]=g_{l},\gamma_{\upsilon }^{j}\left[n\right]=\gamma_{\upsilon }^{j}\right)$, indicating that the LERMS strategy action in the *t*-th time slot is $g_{l},\gamma_{\upsilon }^{j}$. The LERMS action of each generation of data remains unchanged in its occupied time slot, and $g_{l},\gamma_{\upsilon }^{j}$ is set to 0 for the time slot not occupied. Assuming that the number of data packets in each generation does not exceed *B*, we have $\gamma_{j}\in \left\{0,1\right\}$, and let $\gamma_{\upsilon }=\sum_{j=1}^{N_{L}}\gamma_{j}$. Then, we have $\gamma_{\upsilon }\in \Gamma_{\upsilon }\left\{1,\cdots ,N_{L}\right\}$ as $\left\{\gamma_{\upsilon }\in \Gamma_{\upsilon }|0\leq \gamma_{\upsilon }\leq N_{L}C_{\left\{g_{\sigma }\left[t\right]>0\right\}}\right\}$ ($C_{\left\{\cdot \right\}}$ denotes the characteristic function), where $N_{L}$ represents the number of PDU sessions established in LERMS.

### 3.2. Concatenated Encoder Principle

**Figure 4:**
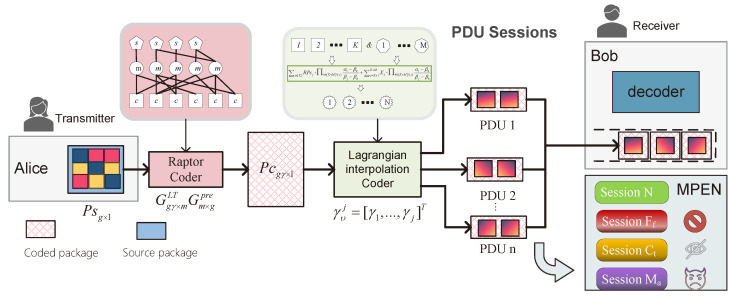
LERMS concatenated encoder scheme.

In this section, we introduce a double-layer concatenated Algorithm 1 for processing data packets within PDU sessions, and the algorithm complexity is O(N·(K+M)·log(K+M)). This encoder scheme combines the advantages of two distinct encoders to ensure reliable, efficient, and secure transmission across multiple disjoint PDU sessions.
**Algorithm 1** Concatenated Encoder **Input:** Read $Ps={\left(Ps_{1},Ps_{2},\cdots ,Ps_{g}\right)}^{T}$ from the queue, $\Omega_{d}=\left(\Omega_{1},\Omega_{2},\cdots ,\Omega_{\max}\right)$, the PDU sessions number $N_{L}$; **Output:** $\bar{P}c$1:$Pc_{g\gamma \times 1}\leftarrow 0_{g\gamma }$;                              ▹ Raptor code encoding procedure2:$G_{g\gamma \times m}^{Raptor}=G_{g\gamma \times m}^{LT}G_{m\times g}^{pre}$;3:$Pc_{g\gamma \times 1}=G_{g\gamma \times g}^{Raptor}Ps_{g\times 1}$;4:$\bar{\mathrm{RP}}c\leftarrow Pc_{g\gamma \times 1}$;           ▹ Packet-level Raptor code encoder output $\bar{\mathrm{RP}}c$5:Generate uniform random matrix $X=\{X_{1},X_{2},\cdots ,X_{M}\}$;6:Split $\bar{\mathrm{RP}}c$ into *K* groups($RPc_{1},RPc_{2},\cdots ,RPc_{K}$);     ▹ Lagrange encoding procedure7:**for** i=1,2,⋯,N **do**8:   $LPc_{i}\leftarrow$$\sum_{j\in [K]}RPc_{j}\cdot \prod_{k\in [K+M]\setminus \{j\}}\frac{\alpha_{i}-\beta_{k}}{\beta_{j}-\beta_{k}}+\sum_{j=K+1}^{K+M}X_{j}\cdot \prod_{k\in [K+M]\setminus \{j\}}\frac{\alpha_{i}-\beta_{k}}{\beta_{j}-\beta_{k}}$; ${\left\{\alpha_{i}\right\}}_{i=1}^{K+M}\cap {\left\{\beta_{j}\right\}}_{j=1}^{K}=\emptyset$9:   $\bar{P}c\leftarrow append\left[\bar{P}c,LPc_{i}\right]$10:**end for**               ▹ Fast Polynomial interpolation11:**return** $\bar{P}c$;

As shown in Figure 4, the LERMS strategy incorporates a concatenated encoder scheme. The first-level encoder, known as the Raptor Encoder, primarily focuses on providing reliability by enhancing the decodability and dependability of data streams transmitted within PDU sessions. The second-level encoder, called the Lagrange Polynomial Encoder, is designed to offer resilient transmission over untrusted paths within the edge network. Through the implementation of the “Double-layer Concatenated Encoder” scheme, we aim to provide a robust encoding solution for PDU sessions. The concatenated encoder principle will be described in detail in two subsections.

#### 3.2.1. Packet-Level Raptor Code Encoder

In this subsection, we present a packet-level Raptor encoding scheme for enhancing the robustness of PDU session transmissions in MPEN. We model these PDU sessions as packet erasure channels, where data packets can either be received entirely or erased. To improve transmission reliability, Ps will be transmitted after the Raptor encoder.

We encode Ps in PDU sessions using a Raptor packet encoder. The LERMS strategy selects *g* data packets for encoding at each time slot. Raptor-encoded data packets Pc are generated through two stages: an outer coder (pre-code) Φ and LT encoder [37] (inner code). The pre-code Φ is a (g,m) block code that generates *m* intermediate coded symbols from *g*Ps. The inner LT encoder generates gγ data packets through ξ(g,m,Ω(x)), where γ represents the encoding redundancy, which is the inverse of the code-rate. The LT encoding matrix is constructed from a predetermined degree distribution $\Omega \left(x\right)=\sum_{d=1}^{d_{\max}}\Omega_{d}x^{d}$, with the degree distribution following a probability distribution $\Omega_{d}=\left(\Omega_{1},\Omega_{2},\cdots ,\Omega_{\max}\right)$ and satisfying $\sum_{d=1}^{d_{\max}}\Omega_{d}=1$. The relationship between the encoder’s input and output is given by $Pc_{g\gamma \times 1}=G_{g\gamma \times m}^{LT}G_{m\times g}^{pre}Ps_{g\times 1}$, where $G_{pre}$ and $G_{LT}$ denote the outer and inner encoding matrices, respectively.

The Raptor coding scheme enhances the reliability of data transmission by mixing data packets. When transmitting over packet erasure channels, it offers higher robustness and resilience. Data transmissions do not require feedback, as the encoding redundancy can be determined based on the channel characteristics. The receiver only needs to receive slightly more than the number of source data packets *g* to complete decoding. Due to the encoding scheme’s data mixing approach, even if some encoded data packets are erased, the entire source data block can still be recovered by continuing to receive encoded data packets.

#### 3.2.2. Encoder for Lagrangian Polynomial Code Multipath Transmission

LCMT performs encoding operations on the output of Raptor encoder, denoted as RPc, to provide safe and reliable transmission over multiple paths, some of which may be untrusted, while protecting data privacy. Let us suppose an MPEN has $N_{L}$ physically isolated paths. LCMT encodes RPc to generate LPc, which is the output of Lagrangian encoder. Each generation of LPc will be split into *K* groups, namely $\left(LPc_{1},LPc_{2},\cdots ,LPc_{k}\right)$, and transmitted to the receiver. Through the reasonable coding method of LCMT, the system aims to tolerate the failure of *S* paths in the MPEN, malicious behavior of *A* paths, and collusion of *T* paths to steal data while still obtaining safe and reliable data transmission. If the data are safely received, we call this transmission scheme realizing the triplet (S,A,T).

To achieve this level of resilience, it is necessary to satisfy the following condition:

(6) $$N_{L}\geq K+T+S+2A$$

At this point, we can say that LCMT can realize the triplet (S,A,T). The significance of this result is that, by adding one path, the link failure resilience can be increased by 1 or the robustness of the malicious behavior path can be increased by 1/2. Furthermore, data privacy can be improved at the same time.

Let us take the transmission of {Pc} as an example, where K=2, N=6, and (S,A,T)=(1,1,1). In this case, {Pc} is split into ${\mathrm{Pc}}_{1}$ and ${\mathrm{Pc}}_{2}$. The key point of LCMT is to select a uniform random matrix X and encode it through Lagrange interpolation polynomial $\left({\mathrm{Pc}}_{1},{\mathrm{Pc}}_{2},X\right)$. The encoding process is given by the following equation:

(7) $$\psi \left(x\right)\overset{\Delta }{\;=}Pc_{1}\frac{\left(x-2)(x-3\right)}{\left(1-2)(1-3\right)}+Pc_{2}\frac{\left(x-1)(x-3\right)}{\left(2-1)(2-3\right)}+X\frac{\left(x-1)(x-2\right)}{\left(3-1)(3-2\right)}$$

To transmit {Pc}, six different values ${\left\{\alpha_{i}\right\}}_{i=1}^{6}$ in the finite field F are determined such that ${\left\{\alpha_{i}\right\}}_{i=1}^{6}\cap \left\{1,2\right\}=\emptyset$. Then, $N_{L}$ PDU sessions transmit $\psi \left(\alpha_{1}\right),\psi \left(\alpha_{2}\right),\cdots ,\psi \left(\alpha_{6}\right)$, where each path transmits the value after interpolation. In other words, the linear combination of ${\mathrm{Pc}}_{1}$ and ${\mathrm{Pc}}_{2}$ is hidden by ξX, where ξ is a nonzero value. Since X is uniformly random, the data privacy of T=1 can be guaranteed. If there is one malicious path (A=1) and one invalid path (S=1), a Reed–Solomon decoder needs to be used at the receiver, and three additional shares of data are required (one additional copy for each invalid path and two additional shares for the malicious path). At the receiving end, $Pc_{1}$ and $Pc_{2}$ can be recovered by computing ψ(1) and ψ(2).

Double-layer concatenated encoder processes downlink data packets from the edge DN to the UE, enabling their transmission through multiple disjoint PDU sessions. By leveraging both Fountain and Lagrange encoders, a concatenated encoder can efficiently and securely handle data packets within the PDU sessions, simultaneously improving transmission reliability and resilience against untrusted path transmissions.

### 3.3. Edge Network with Untrusted Paths

In this section, we analyze the transmission characteristics of MPEN, which is a multipath transmission network consisting of multiple physically disjoint PDU sessions. Our aim is to determine the performance level that can be achieved with the number $N_{L}$ of multiple transmission paths through an analysis of encoding transmission characteristics.

The input and output of MPEN are denoted by X and Y, respectively, while MPEN provides the transmission capability of the edge network. To begin, we define the parameters of the channel model. We assume that MPEN establishes $N_{L}$ paths for the current transmission task, which are classified based on their behavioral characteristics. Specifically, we consider *N* paths that can be transmitted normally, *F* failed sessions that cannot be transmitted within a specified time, *A* malicious transmission paths, and *T* paths that may be compromised by Byzantine attackers or eavesdroppers.

We assume that the input data stream X is evenly divided into *K* sub-packets $x_{1},x_{2},\cdots ,x_{K}$, and these sub-packets are encoded into *N* packets as ${\tilde{x}}_{1},{\tilde{x}}_{2},\ldots ,{\tilde{x}}_{N}$, where K≤N. These encoded packets are distributed across $N_{L}$ PDU sessions for transmission, and the receiver obtains the result $Y=y_{1},y_{2},\cdots ,y_{N}$, where $N=N_{L}$ (Here, we assume that each PDU session has only one single behavioral feature) subject to the following constraints:

(8) $$N_{L}=N+F+A+T$$

Let $\vec{\sum }$ denote the data collection operator and define the receiving vector r, where its *j*-th element $r_{j}$ represents the received data of the *j*-th PDU session. We define four types of receiving situations: normal transmission, represented by $r_{N}$, where $r_{N}={\vec{\sum }}_{n=1}^{N}r_{n}={\vec{\sum }}_{n=1}^{N}{\tilde{x}}_{n}$; failed transmission, represented by $r_{F}$, where $r_{F}={\vec{\sum }}_{f=1}^{F}r_{f}={\vec{\sum }}_{f=1}^{F}\left(0\cdot {\tilde{x}}_{f}\right)$; malicious transmission, represented by $r_{A}$, where $r_{A}={\vec{\sum }}_{a=1}^{A}r_{a}={\vec{\sum }}_{a=1}^{A}\epsilon_{a}\left({\tilde{x}}_{a}\right)$; and Byzantine attack, represented by $r_{T}$, where $r_{T}={\vec{\sum }}_{t=1}^{T}r_{t}={\vec{\sum }}_{t=1}^{T}\epsilon_{t}\left({\tilde{x}}_{t}\right)$. Here, we introduce the functions $\epsilon_{a}$ and $\epsilon_{t}$, which, respectively, represent the influence of malicious transmission channels and Byzantine attack channels on the output.

We assume that the PDU session is a memoryless erasure channel, meaning that the output $r_{i}$ depends only on the input $x_{i}$. Additionally, each data packet has a certain probability of loss, denoted by $\varepsilon_{\upsilon }^{j}=\left[\varepsilon_{1},\cdots ,\varepsilon_{j}\right]$, which represents the erasure probability of different sessions. This loss affects only the receiving result, so we have:

(9) $$Y={\vec{\sum }}_{j=1}^{N_{L}}\varepsilon_{j}r_{j}$$

The above description about MPEN is fully in line with the 3GPP standard’s definition of PDU session [38].

### 3.4. MPEN with Untrusted Path Reliable Function

In this subsection, we analyze the reliability of the two-layer concatenated encoder CMT in the edge network and propose a generalized reliability model. For the enumeration of all possible session state characteristics described in Figure 2, we first employ a $2^{N_{L}}\times N_{L}$ matrix C:

(10) $$C={\left[\begin{array}{cccc} 1 & 1 & \ldots & 0\\ \vdots & \vdots & \ldots & \vdots \\ 1 & 0 & \ldots & 0 \end{array}\right]}^{T}$$

The value 0/1 of the elements in row *i* and column *j* indicates the *i*-th possibility of success/failure at the receiving end through the *j*-th PDU session. After decoding with the Reed–Solomon decoder [39], the malicious path is screened. Error correction and error detection are performed, and abnormal data can be discarded directly; considering that the transmission of this kind of PDU session failed, at this time, the corresponding $c_{i,j}$ is set to 0 to exclude malicious data packets (The defense level against Byzantine attacks is determined during the PDU session establishment and will not be analyzed here).

When transmitting through multiple PDU sessions, we assume that the maximum block error rate and bandwidth guaranteed by GBR QoS [40] for a set of PDU sessions are the same. Based on the delay model described in Section 2, the relationship between packet delivery ratio and transmission delay is established as the cumulative function of the delay probability distribution, called the delay reliability function [30]. Based on end-to-end network monitoring, the delay reliability function is available:

(11) $$F_{LERMS}\left(T,\gamma_{\upsilon }^{j},g\right)=\sum_{i=1}^{2^{N_{L}}}\chi_{i}\prod_{j=1}^{N_{L}}H_{j}\left(T,\gamma_{j}g\right)$$

We consider that $N_{L}$ is at least greater than 3, and the relationship between delay and transmission reliability is given by FLERMS, where

(12) $$\chi_{i}=\left\{\begin{array}{cc} 1, & \mathrm{if}\;\Sigma_{j=1}^{N_{L}}c_{i,j}\gamma_{j}\geq \gamma_{d}\\ 0, & \mathrm{otherwise} \end{array}\right.$$

$g_{i}$ will exclude failed transmissions (i.e., exclude the output of malicious PDU sessions) to ensure that only the correct output of successful decoding is included, and $\gamma_{d}$ is the threshold to ensure successful decoding, with a typical value of 1.05. Hj is defined as

(13) $$H_{j}\left(T,\gamma_{j}g\right)=\left\{\begin{array}{cc} F_{j}\left(T,\gamma_{j}g\right), & \mathrm{if}\;c_{i,j}=1\\ 0, & \mathrm{if}\;c_{i,j}=0 \end{array}\right.$$

Among them, the product of $H_{j}\left(T,\gamma_{j}g\right)$ for $j=1,\cdots ,N_{L}$ appears in the form of a cumulative distribution function (CDF). In the default working mode, the completion of the last data transmission is regarded as the completion of the reliable transmission process.

## 4. Trade-Off Delay-Security for Variable Block-Length LERMS Strategy

### 4.1. The Markov Chain under LERMS Strategies Formulation

Based on the LERMS strategy, we uniformly sample data packets from multiple downlink service flows, and perform joint encoding and transmission. We probabilistically determine the sampling and transmission strategies of different service flows based on the current queue length, and transmit them on the edge network. Specifically, given the queue length $q_{l}\left[n\right]$, l=1,⋯,L, we establish the conditional probability $f_{Q}^{G,\gamma }$ to determine the concatenated encoder coding block length $g_{\sigma }\left[t\right]$, sample from *L* flows, and distribution and transmission strategy $\gamma_{\upsilon }^{j}\left[t\right]$ of *J* paths for the given queue length $q_{l}\left[n\right]$.

(14) $$f_{Q}^{G,\gamma }=P\left\{G\left[t\right]=G,\gamma_{\upsilon }^{j}\left[t\right]=\gamma_{\upsilon }^{j}|Q\left[t\right]=Q\right\}$$

We denote the packet sampling size and buffer queue length of *L* server flows as vectors $G\left[t\right]={\left[g_{1}\left[t\right],g_{2}\left[t\right],\cdots ,g_{L}\left[t\right]\right]}^{T}$ and $Q\left[t\right]={\left[q_{1}\left[t\right],q_{2}\left[t\right],\cdots ,q_{L}\left[t\right]\right]}^{T}$, respectively. The transmission selection and distribution strategy are determined based on the current queue length Q[t]. We denote specific queue lengths and sample sizes by vectors Q and G.

Based on Equation (14), the strategy function of LERMS can be obtained:

(15) $$S=\left\{f_{Q}^{G,\gamma }:Q\in Z^{L},G\in N^{L},1\leq \gamma_{\upsilon }<N_{L},\gamma_{j}\in \left\{0,1\right\}\right\}.$$

Here, the value space of Q and G are $Z^{L}$ and $N^{L}$, respectively, obtained by taking the Cartesian product ${⊙}_{i=1}^{L}Q\left(q_{i}\right)$ and ${⊙}_{i=1}^{L}G\left(g_{i}\right)$. To ensure feasibility of the transmission strategies, we set the value of $f_{Q}^{G,\gamma }$ to 0 for all infeasible strategies G and γ. In other words, if a given combination of packet sampling sizes G and transmission strategies γ is infeasible, its corresponding probability value is forced to 0. To prevent the sender buffer from overflowing or underflowing, the system state $q_{l}$ evolves based on Equation (5). We assume a temporary steady-state condition where no PDU session is being established or released, i.e., $\forall Q\in Z,\sum_{G\in N(Q)}\sum_{\gamma_{\upsilon }^{j}\in \Gamma_{\upsilon }^{N_{L}}\left(G\right)}f_{Q}^{G,\gamma }=1$.

Under the LERMS strategy, we consider the transmission process of downlink server flow in the edge network as a Markov chain, where the queue length Q[t] is the state value of the system. By analyzing the steady-state distribution of the Markov chain, we further analyze how to trade off between latency and reliability. Based on the given strategy S, we first analyze the state transition probability of different queue lengths $\beta_{Q,Q^{\prime }}=P\left\{q_{l}\left[t+1\right]=q_{l}^{\prime }|q_{l}\left[t\right]=q_{l}\right\}$, where Q and $Q^{\prime }$ are the vectors of buffer queue lengths at two consecutive time slots. Specifically, the state transition probability $\beta_{Q,Q^{\prime }}$ can be expressed as

(16) $$\beta_{Q,Q^{\prime }}=\sum_{G\in N^{L}\left(Q\right)}\sum_{\gamma_{\upsilon }^{j}\in \Gamma_{\upsilon }^{N_{L}}\left(G\right)}f_{Q}^{G,\gamma }\prod_{l=1}^{L}\sum_{n=0}^{N}\lambda_{l}^{n}C_{\{\min\left\{{\left(q_{l}-g_{l}\right)}^{*}+n=q_{l}^{\prime }\right\}}$$

where $\lambda_{l}^{n}$ denotes the probability of *n* packet arrivals during single time slot for *l*-th flow. The value range of $Q^{\prime }$ is $Z^{L}$.

Using $\beta_{Q,Q^{\prime }}$, we can determine the steady-state probability $\pi_{S}\left(Q^{\prime }\right)$ for different queue lengths, wherein Q belongs to $Z^{L}$. We can then obtain the Markovian steady-state probability balance equation:

(17) $$\sum_{Q^{\prime }\in Z^{L}\left(Q\right)}\beta_{Q,Q^{\prime }}\pi_{S}\left(Q^{\prime }\right)=\pi_{S}\left(Q\right)$$

where $Z^{L}\left(Q\right)$ is a subset of $Z^{L}$, that contains all possible values of Q under S. The collection branch is expressed as $Z^{L}\left(Q\right)=\left\{Q^{\prime }\in Z^{L}|q_{l}-g_{l}\leq {q^{\prime }}_{l}\leq q_{l}+n,\forall l\right\}$

### 4.2. Constrained Optimization Problem Construction

Based on the steady-state analysis of the state value Q within the edge network, we aim to construct a constrained optimization problem to balance the delay and reliability of multiple downlink flows transmission. Intuitively, joint encoding of multiple service flows’ $P_{s}$ not only improves the coding efficiency but also enhances the security compared to a single service flow. However, it also increases the corresponding queuing delay. Therefore, based on the LERMS strategy, we propose a safe and reliable multi-path transmission encoder strategy. This strategy can effectively utilize the edge network transmission resources and improve the transmission efficiency while satisfying the constraints of reliability functions.

In the constrained optimization problem, we aim to minimize the weighted sum of queuing delays for multiple users while satisfying reliability and system bandwidth constraints. The queuing delay $D_{\mu }^{S}$ is determined based on Little’s Law:

(18) $$D_{\mu }^{S}=\sum_{l=1}^{L}\frac{\mu_{l}}{\lambda_{l}}\sum_{Q\in Z^{L}}\sum_{q^{\prime }\in Z}q^{\prime }\pi_{S}\left(Q\right)C_{\left\{q_{l}=q^{\prime }\right\}}$$

The weight coefficients are represented as $\mu ={\left[\mu_{1},\mu_{2},\ldots ,\mu_{L}\right]}^{T}$, where μ adheres to the conditions of non-negativity and sums up to 1. We can further compute the reliability and bandwidth using the following equations:

(19) $$R^{S}=\sum_{Q\in Z^{L}}\sum_{G\in N^{L}\left(Q\right)}\sum_{\gamma_{\upsilon }^{j}\in \Gamma_{\upsilon }^{N_{L}}\left(G\right)}F_{LERMS}\left(g_{\sigma },\gamma \right)f_{Q}^{G,\gamma }\pi_{S}\left(Q\right)$$

(20) $$W^{S}=\sum_{Q\in Z^{L}}\sum_{G\in N^{L}\left(Q\right)}\sum_{\gamma_{\upsilon }^{j}\in \Gamma_{\upsilon }^{N_{L}}\left(G\right)}W_{S}^{j}\left(g_{\sigma },\gamma_{\upsilon }^{j}\right)f_{Q}^{G,\gamma }\pi_{S}\left(Q\right)$$

We define the optimization variable as $x_{Q}^{G,\gamma }=f_{Q}^{G,\gamma }\pi_{S}\left(Q\right)$, the optimization problem can be formulated as follows:

(21a) $$\begin{array}{cc} \min_{\left\{x_{Q}^{G,\gamma }\right\}}\;\; & \sum_{Q\in Z^{L}}\sum_{G\in N^{L}\left(Q\right)}\sum_{\gamma_{\upsilon }^{j}\in \Gamma_{\upsilon }^{N_{L}}\left(G\right)}D_{\mu }\left(Q\right)x_{Q}^{G,\gamma } \end{array}$$

(21b) $$\begin{array}{cc} s.t.\;\;\; & \sum_{Q\in Z^{L}}\sum_{G\in N^{L}\left(Q\right)}\sum_{\gamma_{\upsilon }^{j}\in \Gamma_{\upsilon }^{N_{L}}\left(G\right)}F_{LERMS}\left(g_{\sigma },\gamma \right)x_{Q}^{G,\gamma }\geq r^{\mathrm{th}} \end{array}$$

(21c) $$\begin{array}{cc} \; & \sum_{Q\in Z^{L}}\sum_{G\in N^{L}\left(Q\right)}\sum_{\gamma_{\upsilon }^{j}\in \Gamma_{\upsilon }^{N_{L}}\left(G\right)}W_{S}^{j}\left(g_{\sigma },\gamma_{\upsilon }^{j}\right)x_{Q}^{G,\gamma }\leq W_{j}^{\mathrm{th}}\\ \; & \sum_{Q^{\prime }\in Z^{L}\left(Q\right)}\sum_{G\in N^{L}\left(Q^{\prime }\right)}\sum_{\gamma_{\upsilon }^{j}\in \Gamma_{\upsilon }^{N_{L}}\left(G\right)}x_{Q^{\prime }}^{G,\gamma }\prod_{l=1}^{L}\lambda_{l}^{q_{l}-{q_{l}}^{\prime }-g_{l}} \end{array}$$

(21d) $$\begin{array}{cc} \; & =\sum_{G\in N^{L}\left(Q\right)}\sum_{\gamma_{\upsilon }^{j}\in \Gamma_{\upsilon }^{N_{L}}\left(G\right)}x_{Q}^{G,\gamma },\;\forall Q\in Z^{L} \end{array}$$

(21e) $$\begin{array}{cc} \; & \sum_{Q\in Z^{L}}\sum_{G\in N^{L}\left(Q\right)}\sum_{\gamma_{\upsilon }^{j}\in \Gamma_{\upsilon }^{N_{L}}\left(G\right)}x_{Q}^{G,\gamma }=1 \end{array}$$

(21f) $$\begin{array}{cc} \; & x_{Q}^{G,\gamma }\geq 0,\;\forall Q\in Z^{L},\;G\in N^{L},\;\gamma_{\upsilon }^{j}\in \Gamma_{\upsilon }^{N_{L}}\left(G\right) \end{array}$$

where $D_{\mu }\left(Q\right)=\Sigma_{l=1}^{L}\frac{\mu_{l}}{\lambda_{l}}q_{l}$.

By solving the optimization problem in Equation (21), we can obtain the minimum average queuing delay under the constraints of reliability and system bandwidth in an untrusted network environment. This allows us to determine the optimal trade-off between latency and reliability. We define the optimal solution of Equation (21) as ${x^{*}}_{Q}^{G,\gamma }$, and we will use the optimal strategy $S^{*}$ to determine the steady-state probability $\pi_{S^{*}}\left(Q\right)$:

(22) $$\pi_{S^{*}}\left(Q\right)=\sum_{G\in N^{L}\left(Q\right)}\sum_{\gamma_{\upsilon }^{j}\in \Gamma_{\upsilon }^{N_{L}}\left(G\right)}{x^{*}}_{Q}^{G,\gamma }$$

among them, we define the lerms optimal strategy as $f^{*}$

(23) $${f^{*}}_{Q}^{G,\gamma }=\left\{\begin{array}{cc} \frac{{x^{*}}_{Q}^{G,\gamma }}{\pi_{S^{*}}\left(Q\right)} & \mathrm{if}\;\pi_{S^{*}}\left(Q\right)>0\\ C_{\{}g={g_{\sigma }}_{Q}^{\max}\} & \mathrm{if}\;\pi_{S^{*}}\left(Q\right)=0, \end{array}\right.$$

where we define ${g_{\sigma }}_{Q}^{\max}=\arg\max_{g\in N^{L}}$. In summary, the optimal strategy $S^{*}$ obtained from the solution of the optimization problem can be used to determine the transmission strategy G[t] and $\gamma_{\upsilon }^{j}\left[t\right]$ based on the current system state Q[t] using the conditional probability $\left\{{f^{*}}_{Q}^{G,\gamma }:G\in N^{L}\left(Q\right),\gamma_{\upsilon }^{j}\in \Gamma_{\upsilon }^{N_{L}}\left(G\right)\right\}$.

### 4.3. Matrix-Based Solving Methods

Considering the exponential growth of the value range of the cache queue Q and transmission policy with the increase in the number of business flows *L* and the number of paths $N_{L}$, this subsection proposes a matrix-based approach to obtain the optimal trade-off for untrusted MPEN transmission. Firstly, we rewrite the linear programming problem in Equation (21) and then, using the unified matrix constraints in Algorithm 2, algorithm complexity is O(L(Z·N+2)), and we automatically generate the LP problem and solve for the LERMS optimal strategy for downlink transmission of multiple service flows.

We represent the optimization variable $x_{Q}^{G,\gamma }$ as a column vector, denoted by x with an index corresponding to the optimization variable $x_{Q}^{G,\gamma }$. The dimension of x is given by

(24) $$\prod_{l=1}^{L}{\left(|Z||N||\Gamma_{\upsilon }^{N_{L}}|\right)}^{l-1}\left(|\Gamma |(|N\right|q_{l}+g_{l})+\gamma_{\upsilon }^{j})+1$$

where |·| denotes the number of elements in the set. We can express Equation (21) in matrix form as follows:
**Algorithm 2** Algorithm to constraints matrix for Equation (21). **Input:** Number of server flows, *L*; Peak flow rate, $N_{\Lambda }$; The upper bounds of g[t], *B*; Number of PDU sessions, $N_{L}$; The probability distribution of Ps, $\lambda_{l}={\left[\lambda_{l}^{0},\lambda_{l}^{1},\cdots ,\lambda_{l}^{N_{\Lambda }}\right]}^{T}$, l=1,⋯,L. Reliability function $F(g_{\sigma },\gamma )$. **Output:** Reliability vector, R; PDU Session Aggregate Maximum Bit Rate vector W; Delay vector, $D_{\mu }$; Matrix for constraints, M.1:$g_{\sigma }\leftarrow 0$, ${\dot{g}}_{\sigma }\leftarrow 1_{\left|Z\right|}⊗{\left[0,1,\cdots ,B\right]}^{T}$, W←0;                    ▹ Generate R, W.2:**for** l=1 to *L* **do**3:   $W\leftarrow W⊗1_{\left|N|\times |Z\right|}$;4:   $g_{\sigma }\leftarrow g_{\sigma }⊗1_{|\dot{g}|}+1_{|g_{\sigma }|}⊗{\dot{g}}_{\sigma }$;5:**end for**6:$W\leftarrow W⊗{\left[0,1,\cdots ,B\right]}^{T}$, $R\leftarrow 0_{|g_{\sigma }|}⊗1_{\left|\Gamma \right|}$;7:**for**$\left\{\gamma_{\upsilon \_i}^{j}\right\}$, i=1 to $2^{N_{L}}$ **do**8:   $R\leftarrow R+F\left(g_{\sigma },\gamma_{\upsilon \_i}^{j}\right)⊗e_{|\Gamma |,i}$;9:**end for**10:$D_{\mu }^{}\leftarrow 0$                        ▹Generate delay vector, $D_{\mu }$.11:**for** l=1 to *L* **do**12:   $D_{l}\leftarrow \frac{\mu_{L-l+1}}{\lambda_{l-l+1}}{\left[0,1,\cdots ,Z\right]}^{T}⊗1_{\left|N\right|}$;13:   $D_{\mu }^{}\leftarrow D_{\mu }^{}⊗1_{|D_{l}|}+1_{|D_{\mu }^{}|}⊗D_{l}$;14:**end for**15:$D\leftarrow D⊗1_{\left|\Gamma \right|}$;16:$\dot{M}\leftarrow 1$, $\ddot{M}\leftarrow 1$, $\tilde{M}\leftarrow 1$;                         ▹ Generate delay vector, M.17:**for** l=1 to *L* **do**18:   **for** q=1 to *Z* **do**19:     ${\dot{M}}_{l,q}\leftarrow 1_{\left|Z|,|N\right|}$, ${\ddot{M}}_{l,q}\leftarrow 1_{\left|Z|,|N\right|}$;20:     **for** all g∈N(q) **do**21:        $M_{l,q,g}\leftarrow$$\left[\begin{array}{c} 0_{q-g,N_{\Lambda }+1};\;\mathrm{diag}\left(1_{1,N_{\Lambda }+1}\right);\;0_{Z-N_{\Lambda }+g-q,N_{\Lambda }+1} \end{array}\right]$;22:        ${\dot{M}}_{l,q}\left(:,g+1\right)\leftarrow M_{l,q,g}\lambda_{L+1-l}$;23:        ${\ddot{M}}_{l,q}\left(:,g+1\right)\leftarrow M_{l,q,g}\lambda_{N_{\Lambda }+1}$;24:     **end for**25:     ${\dot{M}}_{l}\leftarrow \left[{\dot{M}}_{l},{\dot{M}}_{l,q}\right]$, ${\ddot{M}}_{l}\leftarrow \left[{\dot{M}}_{l},{\ddot{M}}_{l,q}\right]$;26:   **end for**27:   $\dot{M}\leftarrow \left(\dot{M}⊗{\ddot{M}}_{l}\right)\left(\ddot{M}⊗{\dot{M}}_{l}\right)$, $\ddot{M}\leftarrow \ddot{M}⊗{\ddot{M}}_{l}$;28:   $\tilde{M}\leftarrow \tilde{M}⊗\left(\mathrm{diag}\left({\left[1_{\left|Z\right|}\right]}^{T}\right)⊗\left({\left[1_{\left|N\right|}\right]}^{T}\right)\right)$29:**end for**30:$M\leftarrow \dot{M}-\tilde{M}$

(25a) $$\begin{array}{cc} \min_{x\geq 0}\;\;\; & D_{\mu }^{T}x \end{array}$$

(25b) $$\begin{array}{cc} s.t.\;\;\; & r^{T}\geq r^{\mathrm{th}} \end{array}$$

(25c) $$\begin{array}{cc} \; & W^{T}\leq W_{j}^{\mathrm{th}}\;\forall j\in \left\{1,2,\cdots ,N_{L}\right\} \end{array}$$

(25d) $$\begin{array}{cc} \; & Mx=0 \end{array}$$

(25e) $$\begin{array}{cc} \; & 1^{T}x=1 \end{array}$$

where 0 and 1 are zero and one vectors, respectively, $f_{Q}^{G,\gamma }$ is the joint encoding and transmission strategy for the given queue length Q, $\beta_{Q,Q^{\prime }}$ is the state transition probability for different queue lengths, $\pi_{S}\left(Q\right)$ is the steady-state probability for the given queue length Q, and $\mu_{l}$ is the weight coefficient for user *l*. $D_{l}$ and $B_{l}$ represent the queuing delay and bandwidth for user *l*, respectively. The maximum values of queuing delay and bandwidth are denoted as $D_{\max}$ and $B_{\max}$, respectively. We define the following vectors: Object delay vector $D_{\mu }$; Reliability vector, R; Bandwidth vector W. We also construct Equation (21d) by the matrix M.

By Algorithm 2, we can automatically obtain Equation (25), where we only need to determine $D_{\mu }$, R, W and M. In Algorithm 2, as shown in lines 10 to 28, we generate the feature matrices for each server flow, namely $D_{\mu }$, $\ddot{M}$, and $\tilde{M}$ for the *l*-th flow. We then construct the target matrix by the Kronecker product ⊗ of these matrices. We define $1_{k}$ and $0_{k}$ as column vectors containing all ones and all zeros, respectively, with *k* items. We also define the sampling vector en,k as an *N*-dimensional column vector, where the *k*-th item is 1. By applying Algorithm 2, we effectively transform Equation (21) as well as Equation (25) into an LP problem in matrix form, allowing us to solve for the optimal strategy for aggregated transmission of downlink multi-service flows in MPEN.

## 5. Numerical Results

In this section, we validate the effectiveness of the LERMS strategy in improving low-latency and highly reliable transmission capabilities in an untrusted edge network environment. To conduct the evaluation, we set up an experimental mobile network and analyze the performance of the proposed LERMS scheme. The core network of LERMS is implemented by enhancing the Free5GC platform [41]. The RCMEN core network is deployed on a laptop equipped with an I7-11800 processor and 16 GB of memory, while the UPF is deployed on a desktop computer with an I7-10700 processor and 32 GB of memory. We simulate the transmission of data by modifying the provided script in the Free5GC.

**Figure 5:**
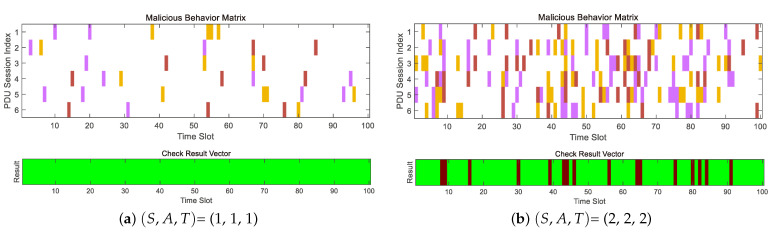
Untrusted edge network with concatenated encoder check results. In the malicious behaviors matrix: link failures are represented in red, malicious tampering in purple, and eavesdropping in yellow.

Firstly, in Figure 5a,b we consider the simulation test of the secure transmission capability of the double-layer encoder. Considering establishing six PDU sessions according to the same situation as described in Section 3.2.2, that is, $N_{L}$ = 6. We will randomly add malicious behavior, including transmission failure link(red), malicious tampering link(purple), eavesdropping link(yellow), i.e., (S,A,T)=(1,1,1). For the concatenated encoder scheme encoding, we use the Reed–Solomon decoder to receive the $\bar{P}c$. To decode, we will identify the decoding result, which can solve the Ps as recognition success, that is, we do not consider the potential safety hazards of Byzantine attackers and eavesdroppers, which is directly regarded as a system capability in the PDU session establishment phase. There is no need to pass the experimental analysis; as shown in Figure 5a, our encoding strategy can provide reliable transmission capabilities in an untrusted network environment. The system has (S,A,T)=(1,1,1) protection capability. As can be seen in Figure 5b, we conducted further tests to evaluate the maximum safety capability. We found that excluding malicious attacks, the maximum capability to recover Ps is (2,0,2). However, as malicious attacks can tamper with data packets, and every tampering of a data packet requires two more data packets for error correction. Thus, in the check matrix, we found that in the time slot corresponding to red, the number of malicious tampering and transmission failures exceeds the range defined by Equation (6). Therefore, it is not possible to provide transmission capability in an untrusted transmission network with $N_{L}=6$.

**Figure 6:**
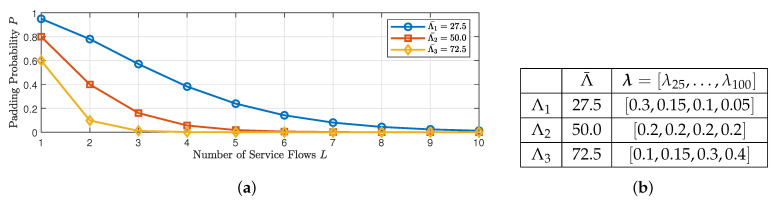
Effectiveness test of SOME-JE strategy to improve LERMS transmission efficiency. (**a**) Relationship between aggregation number *L* and padding probability P. (**b**) Simulation parameter settings.

We analyze the effectiveness of the SOMD-JE strategy, with the aim of coding and ensuring transmission efficiency as well as data encoding security and CMT. The shortest coding block length of Ps is set to 100, and we assume that the arrival probabilities of data packets of different service flows i.i.d, as shown in Figure 6b, while the relationship between the number of aggregated service flows and the probability of padding occurrence is given in Figure 6a. It can be seen that with small $\bar{\Lambda }$, the padding probability is high, and the transmission efficiency of the edge network is low at this time. As the number of aggregated flows *L* increases, P decreases. Therefore, aggregated data flows can significantly reduce the padding probability P; therefore, joint encoding improves transmission efficiency.

**Figure 7:**
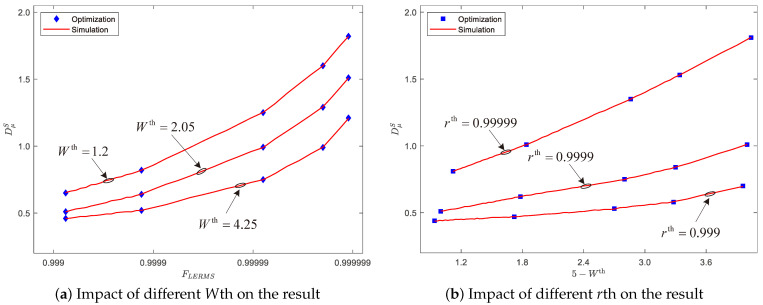
Optimal delay–reliability trade-off curves.

The LERMS strategy is designed to achieve an optimal trade-off between the average queuing delay, available bandwidth, and reliability, with $\varepsilon_{j}$ set to 0.1 for all sessions, resulting in an upper limit of reliability of $1-{\left(0.1\right)}^{6}=0.999999$. As shown in Figure 7, the resulting trade-off curve between latency and reliability is a segmented broken line that matches our theoretical analysis. Figure 7a demonstrates that as the reliability value $F_{LERMS}$ increases, the required queuing delay $D_{\mu }^{S}$ also increases. Moreover, higher available bandwidth *W*th can lead to lower queuing delays $D_{\mu }^{S}$ at a given reliability level *r*. Figure 7b shows that a higher reliability threshold *r*th requires a higher average queuing delay $D_{\mu }^{S}$. These results illustrate the effectiveness of our LERMS strategy in achieving the optimal trade-off between delay and reliability.

## 6. Conclusions and Future Directions

In this paper, we propose a low-latency and highly reliable transmission service for downlink traffic subscribed to edge services in untrusted edge network environments. To address potential failures, malicious tampering, and eavesdropping in the edge network, we introduce an encoder based on Lagrangian interpolation and Raptor double-layer cascading to fully utilize the multipath transmission resources of the edge network and provide secure CMT capabilities. Additionally, we design a variable block length encoding strategy that considers the accumulation of randomly arriving data packets at the sending end and selects an appropriate encoding block length based on queue length state information. We model this decision-making process as a Markov chain and obtain the optimal delay–reliability trade-off through matrix operation methods.

Effective communication and computing management is critical in the context of edge networks, where data is strongly related to the server flow. Currently, CMT is the primary focus, but it is essential to integrate distributed transmission and computing to meet the core requirements of edge networks. By distributing service flows based on computing requirements, we can enable the edge network to be more responsive to the service flow. This approach further enhances the core computing requirements of the edge network for corresponding scenarios, eliminates the single-point vulnerability of central cloud computing, and provides elastic edge network transmission and computing services. Our future research directions include exploring the integration of transmission and computing, multi-session transmission, and multi-DN computing in the edge network.

## Figures and Tables

**Table 1:** Basic notations.

Symbol	Definition
Ps,Pc	The source packets, coded packets.
P,E	The probability, expectation value.
F,N	The finite field, the set of natural numbers.
$\vec{\sum }$	The data collection operator for receiver.
*T*	The time span of timeslot.
Ω(x)	The degree distribution.
*g*	The block-length of codes.
g[t],$\gamma_{\upsilon }^{j}\left[t\right]$	The encoding action within t-th timeslot.
$d^{\mathrm{th}}$	The constraints of delay.
*B*	The upper bounds of g[t].
Z	The transmitting side queue buffer size.
ϕ	The size of each data packet.
$\varepsilon_{\upsilon }^{j}=\left[\varepsilon_{1},\cdots ,\varepsilon_{j}\right]$	The erasure probability of *j* sessions.
$\Lambda_{l}\left[t\right]$, λ=	The number Ps arrivals within *t*-th timeslot for user *l*.
$\left[\lambda_{l}^{0},\lambda_{l}^{1},\cdots ,\lambda_{l}^{N}\right]$,	The probability distribution of $\Lambda_{l}\left[t\right]=n$.
$N_{L}$	The number of disjoint PDU sessions established in MPEN.

## Data Availability

The 5G Core Network that support this study are available from https://github.com/free5gc/free5gc (accessed on 1 February 2021).

## Appendix A. Multiple PDU Sessions Transmission Procedure for Aggregation Data of Downlink Multi-Service Flow

In the scenario of multiple-device single-owners service flow aggregation transmission, we propose a procedure to support data aggregation over multiple PDU sessions. This is in addition to the existing physical isolation multi-PDU session establishment [27]. Currently, the core network does not support the joint transmission of multiple service flows. In this context, we propose the use of multi-PDU sessions to transmit the downlink data flow of edge computing services. This supports the transmission of aggregated traffic streams through physically isolated PDU sessions on a per-session basis.

1. The DN initiates the PDU session establishment request by sending a PDU Session Establishment Request message, which includes the requested DNN (identifier of the data flow from the core network to the UE), PDU session type, etc. The proposed procedure establishes a group of physically isolated multi-PDU sessions and assigns a session ID to this group of PDU sessions.
2. All devices within the same aggregated service flow must be assigned the same session ID.
3. Anchor UPF-A can identify the aggregated service flow through a specific session ID, facilitating unified management within the core network.
4. The local DN (MEC nodes) must perform the operation of inserting tags. Before the service flow is aggregated, the data owner is identified by adding a unique tag to the data packet.
5. After the device receives the aggregated data packet and completes decoding, it distinguishes the data according to the tag and extracts the data it needs. The rest of the data can be used to verified untrusted paths or discarded directly.
